# Identification of two linear B-cell epitopes from West Nile virus NS1 by screening a phage-displayed random peptide library

**DOI:** 10.1186/1471-2180-11-160

**Published:** 2011-07-06

**Authors:** En-Cheng Sun, Jian-Nan Ma, Ni-Hong Liu, Tao Yang, Jing Zhao, Hong-Wei Geng, Ling-Feng Wang, Yong-Li Qin, Zhi-Gao Bu, Yin-Hui Yang, Ross A Lunt, Lin-Fa Wang, Dong-Lai Wu

**Affiliations:** 1 The Key Laboratory of Veterinary Public Health, Ministry of Agriculture, State Key Laboratory of Veterinary Biotechnology, Harbin Veterinary Research Institute, Chinese Academy of Agricultural Sciences, 427 Maduan Street, Nangang District, Harbin 150001, PR China; 2 Graduate School of Chinese Academy of Agricultural Sciences, 12 Zhongguancunnandajie, Haidian District, Beijing 100081, PR China; 3 Beijing Institute of Microbiology and Epidemiology, 20 Dongda Street, Fengtai District, Beijing 100071, PR China; 4 CSIRO Livestock Industries, Australian Animal Health Laboratory, Private Bag 24, Geelong, Victoria 3220, Australia

## Abstract

**Background:**

The West Nile virus (WNV) nonstructural protein 1 (NS1) is an important antigenic protein that elicits protective antibody responses in animals and can be used for the serological diagnosis of WNV infection. Although previous work has demonstrated the vital role of WNV NS1-specific antibody responses, the specific epitopes in the NS1 have not been identified.

**Results:**

The present study describes the identification of two linear B-cell epitopes in WNV NS1 through screening a phage-displayed random 12-mer peptide library with two monoclonal antibodies (mAbs) 3C7 and 4D1 that directed against the NS1. The mAbs 3C7 and 4D1 recognized phages displaying peptides with the consensus motifs LTATTEK and VVDGPETKEC, respectively. Exact sequences of both motifs were found in the NS1 (_895_LTATTEK_901 _and _925_VVDGPETKEC_934_). Further identification of the displayed B cell epitopes were conducted using a set of truncated peptides expressed as MBP fusion proteins. The data indicated that _896_TATTEK_901 _and_925_VVDGPETKEC_934 _are minimal determinants of the linear B cell epitopes recognized by the mAbs 3C7 and 4D1, respectively. Antibodies present in the serum of WNV-positive horses recognized the minimal linear epitopes in Western blot analysis, indicating that the two peptides are antigenic in horses during infection. Furthermore, we found that the epitope recognized by 3C7 is conserved only among WNV strains, whereas the epitope recognized by 4D1 is a common motif shared among WNV and other members of Japanese encephalitis virus (JEV) serocomplex.

**Conclusions:**

We identified TATTEK and VVDGPETKEC as NS1-specific linear B-cell epitopes recognized by the mAbs 3C7 and 4D1, respectively. The knowledge and reagents generated in this study may have potential applications in differential diagnosis and the development of epitope-based marker vaccines against WNV and other viruses of JEV serocomplex.

## Background

West Nile virus (WNV) is the etiological agent of West Nile fever (WNF), an important mosquito-borne disease widely prevalent in Africa, Europe, Russia, the Middle East, India, Australia and also in North America since 1999 [1]. WNV has expanded its geographic range since the first identification of WNV cases in the United States in 1999, and only in 2010, 981 human cases of WNF were reported in the United States [2]. WNV is serologically classified into the Japanese encephalitis virus (JEV) serocomplex, including JEV, Saint-Louis encephalitis virus (SLEV), Murray Valley fever virus (MVEV) and Kunjin virus, all of which are responsible for severe encephalitis in humans and related animals [3,4].

The 10.7-kilobase genome of WNV encodes a single polyprotein, which is cleaved into three structural proteins (C, prM/M, and E) and seven nonstructural proteins (NS1, NS2A, NS2B, NS3, NS4A, NS4B and NS5) by both virus- and host-encoded proteases. The seven nonstructural proteins (glycoprotein NS1 and NS2A, protease cofactor NS2B, protease and helicase NS3, NS4A, NS4B and the polymerase NS5) associate with viral RNA to form the replication complex [5]. NS1 is a 48-Kd glycoprotein containing 12 invariant cysteine residues. The antigenic variability of the NS1 provides a useful mechanism to differentiate closely related flaviviruses [6]. NS1 is also inserted into the lumen of the endoplasmic reticulum via a signal peptide that is cleaved cotranslationally by a cellular signalase to generate the mature N terminus of the protein [7]. Within infected cells, NS1 is believed to function as a cofactor in viral RNA replication, and specific amino acids substitutions in NS1 can attenuate viral RNA accumulation [8].*In vivo*, highly circulating levels of the Dengue virus (DENV) NS1 early in Dengue illness correlated with the development of Dengue hemorrhagic fever and other severely associated diseases [9].

The diagnosis of WNV and associated diseases has long been a challenge, especially in the field of differential diagnosis. Assays employing reverse transcription-polymerase chain reaction (RT-PCR) are able to differentiate closely related viruses, but these assays can only be applied to specimens containing circulating virus or viral RNA. Serological tests for WNV infections mainly include the neutralization test, the hemagglutination-inhibiting test, the enzyme-linked immunosorbent assay (ELISA) and the immunofluorescence assay (IFA) [10]. Among these tests, the neutralization test is recognized as the "gold standard" and provides the highest specificity. However, neutralization assay requires paired acute- and convalescent-phase serum specimens, and involves manipulation of live virus which requires a high level of biocontainment. The use of the IFA as a diagnostic tool is also limited by practical issues related to biosafety. The ELISA has also been used to detect immunoglobulin M (IgM) antibodies that specifically react with WNV antigens. However, these tests may be confounded by the potential cross-reactivity of antibodies with other members of the JEV serocomplex or other flaviviruses [11-13], especially in regions where several flaviviruses coexist [14]. In 1995, Hall *et al *developed an assay in which antibodies against immunodominant epitopes in NS1 of MVEV and Kunjin viruses were used to define targets for a blocking ELISA. This assay was used to detect virus-specific antibodies in sentinel animal sera, and confirmed that NS1 could be used as a target protein to differentiate viruses in the JEV serocomplex [15]. In a recent study, an epitope-blocking ELISA based on a WNV NS1-specific mAb was established and used to differentiate WNV from JEV infections in horses and to detect natural infections among vaccinated populations [16-19].

Phage display describes an *in vitro *selection technique in which a peptide or protein is genetically fused to a coat protein of a bacteriophage, resulting in displaying of the fused peptide or protein on the exterior of the phage virion. Phage display library can consist of either a random peptide library or a gene-targeted library, and thus provides a powerful and economic technique for epitope identification. This technology can identify amino acids in protein antigens which are critical for antibody binding and, further, the definition of peptide motifs that are both structural and functional mimotopes of both protein and non-protein antigens [20,21]. Several studies have reported the usefulness of phage-display applications for mapping epitopes of flaviviruses [22-25].

The aim of our study was to identify WNV-specific and/or JEV serocomplex-specific B-cell epitopes in NS1 using phage display technology. The information provided by this study will facilitate the development of diagnostic tools for the specific serological diagnosis of WNV infection, and will contribute to the rational design of vaccines by furthering understanding of the antigenic structure of NS1.

## Results

### Production of recombinant NS1

**Figure 1:**
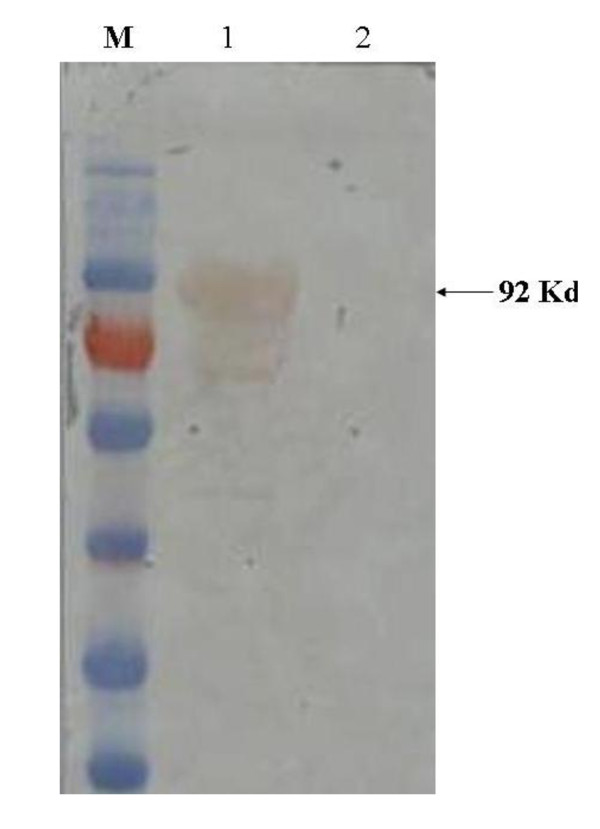
**WNV-positive equine sera recognize recombinant NS1**. Binding of antibodies from WNV-positive equine serum to recombinant NS1 (lane 1) and MBP-tag (lane 2) by Western blot. M, PageRuler™ Prestained Protein Ladder (Fermentas, Canada).

Recombinant WNV NS1 was successfully expressed in *E. coli *TB1 cells, predominantly as soluble protein, after induction with isopropyl β-D-1-thiogalactopyranoside (IPTG). The recombinant protein was recognized by WNV-positive equine serum in Western blot (WB) (Figure 1, lane 1).

### Production and characterization of NS1-specific mAbs

**Figure 2:**
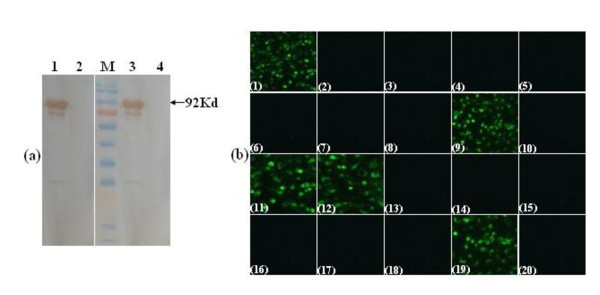
**Reactivity of mAbs with recombinant NS1 and C6/36 cells infected with flaviviruses**. (a) Western blot analysis of mAbs 3C7 (lanes 1, 2) and 4D1 (lanes 3, 4) against recombination NS1 (lane 1, 3) and MBP-tag (lane 2, 4). M, PageRuler™ Prestained Protein Ladder (Fermentas, Canada). (b) Pattern of immunofluorescence produced by anti-NS1 mAbs on antigen slides which were prepared on porous slides using C6/36 cells infected with different flaviviruses. Panels 1-8: reactivity of mAb 3C7 with cells infected with WNV (panel 1), JEV (panel 2), DENV1 (panel 3), DENV2 (panel 4), DENV3 (panel 5), DENV4 (panel 6), YFV (panel 7), and TBEV (panel 8). Panels 11-18: reactivity of mAb 4D1 with cells infected with WNV (panel 11), JEV (panel 12), DENV1 (panel 13), DENV2 (panel 14), DENV3 (panel 15), DENV4 (panel 16), YFV (panel 17), and TBEV (panel 18). Panels 9 and 19 are positive controls which used WNV-positive mouse serum. Panels 10 and 20 are negative controls which used WNV-negative mouse serum.

Purified protein was used to immunize BALB/c mice. After cell fusion and screening, several hybridoma cell lines were obtained which produced NS1-specific mAbs. Among them two cell lines were selected for their strongest reactivity against recombinant NS1 using indirect ELISA (data not shown), WB (Figure 2a), and against native NS1 in IFA using WNV antigen slides (Figure 2b). Further characterization of the specificity of the two mAbs by IFA, demonstrated that the mAb 3C7 reacted with WNV, but did not react with JEV, DENV1-4, Yellow fever virus (YFV) and Tick-borne encephalitis virus (TBEV), whereas mAb 4D1 reacted with both WNV and JEV, but did not react with other non-JEV serocomplex flaviviruses (Figure 2b).

The subtypes of the two mAbs were determined using the Mouse MonoAb-ID Kit (HRP) according to the manufacturer's instructions. It was shown that the heavy chain of 3C7 and 4D1 was IgG1 and the light chain was λ type. Antibody titers of culture supernatants of the two hybridoma cell lines and the ascites prepared with them were measured by indirect ELISA. Antibody titers of the culture supernatants of mAbs 3C7 and 4D1 were 1:256 and 1:512, respectively; and those of the ascites were 1:512,000 and 1:1,024,000, respectively.

### Phage enrichment by biopanning

Preparations of mAbs 3C7 and 4D1 were purified to >90% (as determined by SDS-PAGE) and used to define peptide binding motifs by screening a phage-displayed 12-mer peptide library. A dramatic enrichment of 3C7 and 4D1 antibody-reactive phages was achieved with three sequential rounds of biopanning. As a measure of enrichment, we calculated output-to-input ratios following each round of selection with each mAb. The output-to-input ratio is defined as the percentage of plaque-forming phages remaining after elution from the mAbs. The output-to-input ratios of the three rounds of biopanning were 0.00016%, 0.023% and 0.88% for the mAb 3C7, and 0.00018%, 0.023% and 0.89% for the mAb 4D1, indicating significant enrichment of antibody reactive phage clones.

### Epitope prediction

**Figure 3:**
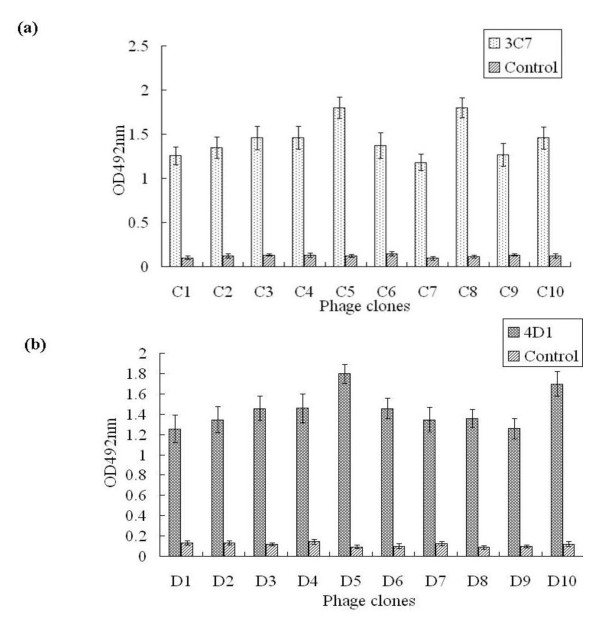
**Monoclonal antibody recognition of clones selected from the phage displayed peptide library**. Ten clones selected after three rounds of biopanning from phage display peptide library were tested for binding to each respective mAb by phage ELISA. (a) C1-C10 for binding to mAb 3C7; (b) D1-D10 for binding to mAb 4D1; in both cases, the anti-porcine IFN-γ mAb served as negative control.

**Figure 4:**
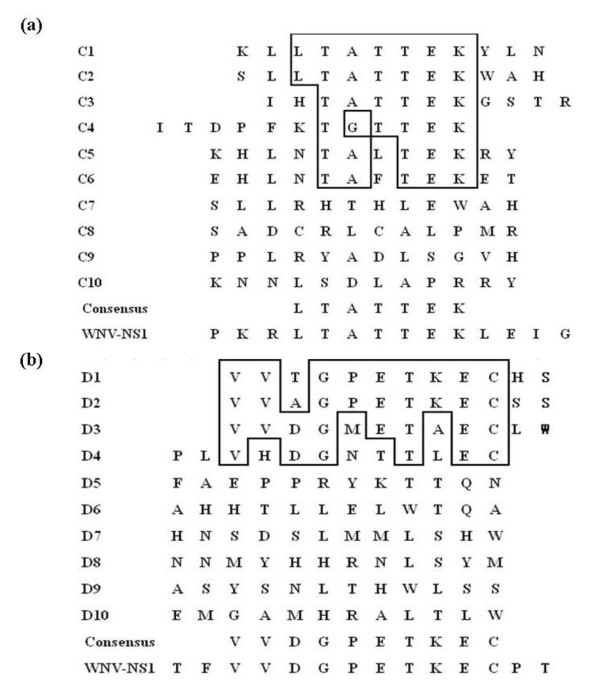
**Alignment of 12-mer peptide sequences from ELISA-positive clones defined the linear epitopes for the mAbs 3C7 (a) and 4D1 (b)**. The peptides inserted from ten phage clones that reacted with the mAbs 3C7 and 4D1 were aligned. Conserved amino acid residues are boxed and consensus sequence motifs were provided below the alignments. The matching sequences _895_LTATTEK_901 _and _925_VVDGPETKEC_934 _in WNV NS1 are provided at the bottom of alignment for comparison.

Phage ELISA results showed that the selected ten phage clones for every mAb (C1-C10 for 3C7 and D1-D10 for 4D1) demonstrated specific reactivity (OD492 nm > 1.0) in comparison to a negative control of irrelevant specific mAb, the anti-porcine interferon-γ (IFN-γ mAb (OD492 nm < 0.20) (Figure 3). By sequencing to determine the insert sequences, alignment indicated that six 3C7-reactive clones (C1-6) displayed a consensus sequence of LTATTEK. Similarly, four 4D1-reactive clones (D1-4) revealed another consensus sequence of VVDGPETKEC. These consensus sequence motifs are identical to WNV NS1 sequences _895_LTATTEK_901 _and _925_VVDGPETKEC_934_, respectively (Figure 4).

### Identification of the displayed epitopes by WB using expressed polypeptides

**Figure 5:**
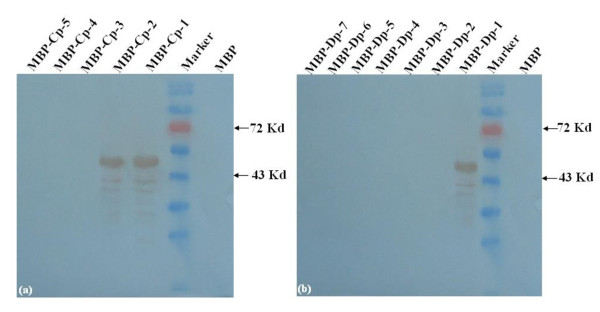
**Reactivity of the recombinant MBP-fusion proteins containing wild-type and truncated motifs with mAbs 3C7 (a) and 4D1 (b)**. M, PageRuler™ Prestained Protein Ladder (Fermentas, Canada). The MBP-fusion proteins including the polypeptides: MBP-Cp-1(LTATTEK); MBP-Cp-2 (TATTEK); MBP-Cp-3(LTATTE); MBP-Cp-4(ATTEK); MBP-Cp-5(LTATT); MBP-Dp-1(VVDGPETKEC); MBP-Dp-2(VDGPETKEC); MBP-Dp-3(VVDGPETKE); MBP-Dp-4(DGPETKEC); MBP-Dp-5(VVDGPETK); MBP-Dp-6(GPETKEC); MBP-Dp-7(VVDGPET).

For further epitope determination, a series of wild-type and truncated peptides deriving from LTATTEK (3C7) and VVDGPETKEC (4D1) were produced and subjected to WB. Results were shown that MBP-Cp-1 (MBP-fused polypeptide containing Cp-1 peptide: LTATTEK) and MBP-Cp-2 (MBP-fused polypeptide containing Cp-2 peptide: TATTEK) were recognized by mAb 3C7, and only MBP-Dp-1 (MBP-fused polypeptide containing Dp-1 peptide: VVDGPETKEC) was recognized by mAb 4D1, whereas all other peptides were unable to react with the respective mAb (Figure 5). These data define TATTEK and VVDGPETKEC as the linear epitopes recognized by 3C7 and 4D1, respectively.

### Reactivity of WNV/JEV-positive sera with the identified NS1 epitopes

**Figure 6:**
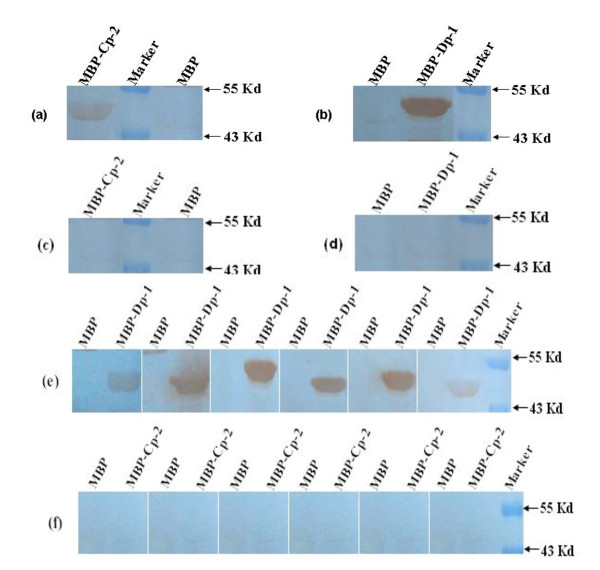
**Reactivity of recombinant MBP-fusion proteins containing epitopes TATTEK (MBP-Cp-2) and VVDGPETKEC (MBP-Dp-1) with WNV/JEV-positive equine serum by WB**. MBP alone or MBP fused with the TATTEK (MBP-Cp-2) and VVDGPETKEC (MBP-Dp-1) peptides were evaluated by WB for reactivity with antibodies in WNV/JEV-positive equine serum. MBP-fused proteins containing the two epitopes reacted with WNV-positive equine serum (Fig. 6 a, b) and WNV-negative equine serum (Fig. 6 c, d). The polypeptide Dp-1 and Cp-2 reacted with six JEV-positive equine sera, respectively (Fig. 6 e and f). M: PageRuler™ Prestained Protein Ladder (Fermentas, Canada).

Recombinant proteins containing the two epitopes were recognized by WNV-positive equine serum in WB (Figure 6a, b), whereas they were not recognized by WNV-negative control equine serum (Figure 6c, d). Further cross-reaction detection showed the polypeptide Dp-1 (VVDGPETKEC) could react with six JEV-positive equine sera (Figure 6e), but Cp-2 (TATTEK) was not recognized by any JEV-positive equine serum (Figure 6f). This was further confirmed by ELISA (data not shown). These data indicate that the two peptides are antigenic in horses.

### Sequence similarity and prediction of cross-reactivity

**Figure 7:**
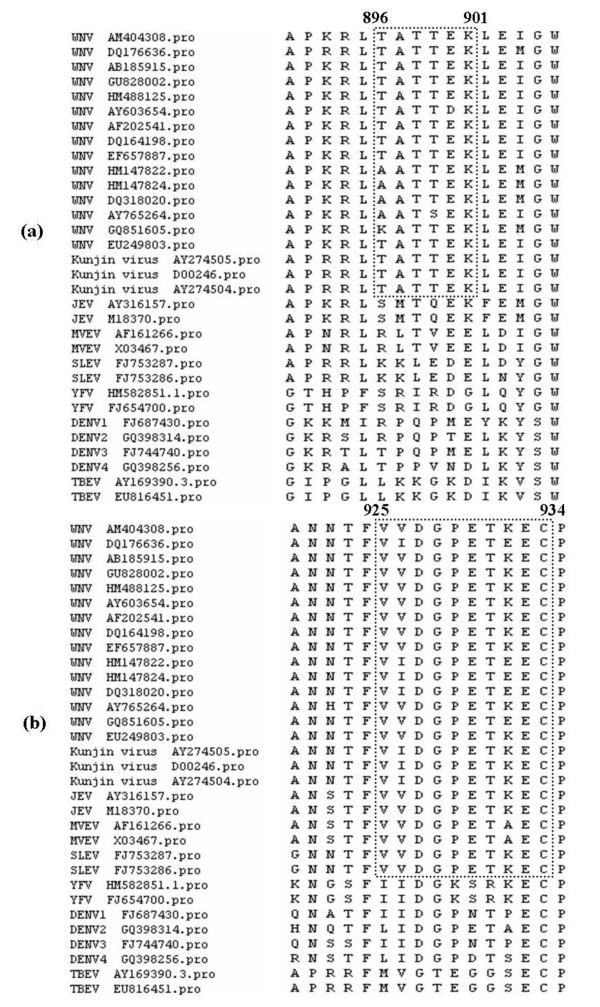
**Alignment of the 3C7 and 4D1 linear epitopes with the NS1 sequence of WNV and other flaviviruses**. A total of 18 WNV strains (12 WNV lineage 1 strains including 3 Kunjin virus strains and other four lineages of WNV strains: lineage 2 (HM147822, HM147824, DQ318020), lineage 3 (AY765264), lineage 4 (GQ851605) and lineage 5 (EU249803)) and 14 associated flavivirus virus strains were used in the analysis. The sequence motif recognized by each mAb was boxed.

To assess the degree of conservation of the linear epitopes recognized by the 3C7 and 4D1 mAbs, we analyzed the NS1 amino acid sequences from WNV isolates including Kunjin virus strains, and other members of the family *Flaviviridae*. Analysis of NS1 sequences from 18 different WNV isolates indicated that the 3C7 epitope, TATTEK is highly conserved among WNV lineage 1 strains including Kunjin virus strains and WNV lineage 5 strains (EU249803; Figure 7a). Limited amino acid mutations were present in WNV lineage 2, 3 and 4 strains (Figure 7a). Notably, the sequence motif is not conserved among other members of the family *Flaviviridae*, including other viruses of the JEV serocomplex (Figure 7a). We also performed sequence alignments for the minimal linear epitope recognized by the 4D1 mAb. The motif VVDGPETKEC was a common epitope of JEV serocomplex members, including WNV, JEV, MVEV and SLEV, but was absent of non-JEV serocomplex members of the family (Figure 7b).

## Discussion

NS1 is an important non-structural protein of flaviviruses. The impact of NS1 activity on flavivirus RNA replication, host recognition of virus-associated molecular patterns and anti-viral protective immunity has been well documented [26-29], as it has the importance of antibodies generated against NS1. Studies have demonstrated that the passive administration of NS1-specific mAbs or active immunization with the NS1 gene or protein confers protection from lethal flavivirus challenge [30,31]. Such protective effect could even be observed when using NS1 produced by *E. coli *[32,33]. These results demonstrate that immune responses specifically directed against NS1 play important roles in conferring immune protection during infection with flaviviruses.

MAbs with well-defined epitopes provide an experimental platform for studying antigen structure, and developing diagnostic reagents and therapeutics for pathogen control [34-38]. Precise analysis of the epitopes in NS1 is important for understanding the mechanism of NS1-mediated protection. In recent years, epitope-based marker vaccine has increasingly received attentions. By inserting confirmed epitopes into a target protein to immunize animals, diagnostic methods based on the detection of antibodies generated against the inserted epitopes could be developed to investigate whether the generation of detected antibody was a result of vaccination or natural infection. NS1 is antigenic and elicits the generation of protective antibodies. Identifying linear epitopes in NS1 would contribute to developing epitope markers and epitope-based marker vaccines. There are a few reports of mapping epitopes in NS1 of DENV [39-41], TBEV [29] and JEV [42]. In the case of WNV, epitope mapping has been exclusively focused on the viral envelope (E) glycoprotein [43,44]. To our knowledge, there has been no report mapping epitopes in the WNV NS1.

In our current study, a panel of NS1-specific mAbs was produced using soluble recombinant NS1 expressed in *E. coli*. Two of the strongest reactive mAbs were selected for further characterization based on IFA results. The mAb 3C7 only reacted with WNV while the mAb 4D1 reacted with both WNV and JEV, but not other non-JEV serocomplex flaviviruses, such as DENV1-4, YFV and TBEV.

The epitopes recognized by the two mAbs were determined using phage display technology, which has been demonstrated to be a powerful and high-throughput tool for the rapid mapping of epitopes [21,22,25]. Two consensus peptide sequences corresponding to_896_TATTEK_901 _and_925_VVDGPETKEC_934 _were identified. These peptides were also recognized by WNV-positive equine serum, but not WNV-negative equine serum, indicating that the identified epitopes are antigenic in the context of bona fide WNV infection. Although, our laboratory only has one WNV-positive equine serum sample from CSIRO Australian Animal Health Laboratory, we tested six JEV-positive equine sera for reactivity against the identified linear epitopes. None of the JEV-positive equine sera reacted with the 3C7 epitope, whereas the 4D1 epitope reacted with all JEV-positive equine sera by WB. Importantly, sequence alignment confirmed our experimental data, as the epitope recognized by 3C7 was completely conserved among WNV lineages 1 (including Kunjin strains) and 5, moderately conserved in WNV lineages 2, 3 and 4, but not conserved in JEV. The potential cross-reactivity of 3C7 with WNV lineages 2, 3 and 4, where the first position of the peptide was mutated, needs to be determined. The 4D1 epitope is conserved in JEV serocomplex members with the exception of one amino acid (amino acid position 926, V→I). However, further evaluation revealed that the V→I mutation does not affect the reactivity of 4D1 mAb (data not shown).

The high degree of antibody cross-reactivity generated among animals infected with flaviviruses has been a diagnostic challenge, and this limitation is apparent for members of JEV serocomplex when using the gold standard neutralization test [12]. This is largely due to the presence of highly conserved and immunodominant epitopes in the viral E glycoprotein that are responsible for eliciting cross-reactive serum antibodies after infection [44]. Thus, it is remarkable that we have identified a WNV-specific epitope in NS1 since such an epitope has great potential to improve WNV serological diagnostic tests and contribute to the development of epitope-based marker vaccines.

## Conclusions

The TATTEK and VVDGPETKEC are WNV NS1 specific linear B-cell epitopes recognized by the mAbs 3C7 and 4D1, respectively. The knowledge and reagents generated in this study may have applications in the differential diagnosis of viral infection and in the development of epitope-based marker vaccines against WNV and other viruses of JEV serocomplex.

## Methods

### Cell lines, plasmids, sera and viruses

The myeloma cell line SP2/0 was cultured in Dulbecco's modified Eagle's medium (DMEM, Invitrogen) in humidified 5% CO_2 _atmosphere at 37°C. All culture media were supplemented with 10%, 56°C 30 min heat-inactivated fetal bovine serum (GIBCO, Invitrogen) and antibiotics (0.1 mg ml^-1 ^of streptomycin and 100 IU ml^-1 ^of penicillin). The plasmids containing the WNV NY99 genome (GenBank AY842931.3) and pMAL™-C2x (New England Biolabs, Inc., USA) were maintained in our laboratory. WNV-positive/negative mouse sera were obtained from Beijing Institute of Microbiology and Epidemiology, and the WNV-positive/negative equine sera were acquired from the CSIRO Australian Animal Health Laboratory (AAHL). The flaviviruses strains (DENV-1, D1-ZJ-57; DENV-2, D2-43; DENV-3, D3-80-2; DENV-4, D4-B5; JEV, SA-14-14-2, GenBank AF315119.1; TBEV, Senzhang, GenBank AY182009.1; WNV, Chin-01, GenBank AY490240.2; and YFV, 17D/tiantan, GenBank FJ654700.1) used in this study were obtained from Beijing Institute of Microbiology and Epidemiology.

### Expression of recombinant NS1

The full-length NS1 coding sequence was amplified using the primers WNVNS1-*EcoRI*-2470F (5'-GTA*GAATTC*GACACTGGGTGTGCCATAG-3') and WNVNS1-*XhoI*-3526R (5'-TGA*CTCGAG*CATTCACTTGTGACTGCAC-3'). These primers were designed according to the sequence of WNV NY99 strain (GenBank AY842931.3) and contained *EcoR *I and *Xho *I sites (shown in italics) to facilitate directional cloning into the pMAL™-C2x expression vector following amplification, agarose gel purification and restriction enzyme digestion. The recombinant plasmid was verified by restriction enzyme digestion and DNA sequencing, then it was transformed into *E. coli *TB1 (Takara) cells for expression. After several hours of shaking, when the optical density (OD600 nm) is up to 0.5~0.7, IPTG (Pharmacia Biosciences) was added to a final concentration of 0.5 mM into Rich medium (per liter include: 10 g tryptone, 5 g yeast extract, 5 g NaCl, 2 g glucose, autoclave; add sterile ampicillin to 100 μg ml^-1^) and a further 10 h incubation at 16°C in agitation was performed. Then bacteria were pelleted at 9000 g for 10 min at 4°C and lysed by sonication in Column Buffer (20 mM Tris-HCl, 200 mM NaCl, 1 mM EDTA), sodium dodecyl sulfate-polyacrylamide gel electrophoresis (SDS-PAGE) was carried out to analyze the maltose-binding protein (MBP) tagged recombinant NS1, and the reactivity identified by WB using WNV-positive/negative equine serum. For WB, briefly, pMAL-NS1 recombinant protein and pMAL™-C2x expressed MBP-tag were subjected to electrophoresis on 12% SDS-PAGE after reduction with dithiothreitol (DTT) at 100°C for 5 min, then samples were transferred to a nitrocellulose membrane, nonspecific antibody binding sites were blocked with 5% skimmed milk powder in PBST overnight at 4°C. The membrane was incubated with WNV-positive/negative equine serum as the primary antibody at a 1:100 dilution, after incubation, each was washed five times with PBST, then it was treated with HRP-conjugated rabbit anti-equine secondary antibodies (LICOR Biosciences). The color was developed using 3,3'-diaminobenzidine tetrahydrochloride (DAB) and stopped by rinsing in deionized water followed by drying the membrane.

### Preparation and identification of mAbs against NS1

Recombinant NS1 fused with MBP-tag was produced in *E. coli *as soluble in the cell lysate following IPTG induction. For preparation of immunogen, the soluble NS1 was purified by Amylose Resin according to pMAL™ Protein Fusion and Purification System, Version 5.01 (New England Biolabs, Inc., USA). Purified NS1 was used for immunization. Hybridoma cells secreting anti-NS1 antibodies were generated according to standard procedures [45]. Briefly, six-week-old female BALB/c mice were immunized subcutaneously with purified NS1 emulsified with an equal volume of Freund's complete adjuvant (Sigma, St. Louis, MO, USA). Two booster injections containing purified NS1 with equal volume of Freund's incomplete adjuvant were given at 2-week intervals. The final immunization, purified NS1 without adjuvant was given intraperitoneally. Three days after the final dose, mice were euthanized and spleen cells were harvested and fused with SP2/0 myeloma cells at 5-10:1 ratio using polyethylene glycol (PEG 4000, Sigma). Hybridoma cells were seeded into 96-well plates and selected in HAT medium (DMEM containing 20% fetal bovine serum, 100 ug ml^-1 ^streptomycin, 100 IU ml^-1 ^penicillin, 100 mM hypoxanthine, 16 mM thymidine and 400 mM aminopterin), and after 5 days, the medium was removed and replaced with fresh HT-DMEM medium. After HAT/HT selection, culture supernatants of surviving clones were screened for reactivity and specificity by indirect ELISA, WB and IFA.

The ELISA was described previously [46]. Briefly, microplates were sensitized at 4°C overnight with affinity-purified WNV-NS1 antigen at 100 ng ml^-1^. The sensitized plates were incubated with culture supernatants from hybridoma cells at 37°C for 1 h, with HRP-conjugated goat anti-mouse secondary antibodies (LICOR Biosciences) at a 1:4,000 dilution at 37°C for 1 h, followed by color development with substrate solution containing *o*-phenylenediamine (OPD). WB was performed as described above, but the primary antibodies were the mAbs supernatant and HRP-conjugated goat anti-mouse secondary antibodies were used.

The IFA results were supplied by Beijing Institute of Microbiology and Epidemiology. WNV, JEV, DENV1-4, YFV and TBEV antigen slides were prepared on porous slides using WNV, JEV, DENV1-4, YFV and TBEV infected and uninfected C6/36 cells. Cell suspensions were dripped onto slides, fixed using acetone, air dried and stored at -20°C. Next, anti-NS1 mAbs supernatant and WNV-, JEV-, DENV1-4-, YFV- and TBEV-positive/negative mouse sera (working dilution was 1:100) (positive/negative control) were incubated on acetone-fixed antigen slides for 2 h. A FITC-conjugated goat anti-mouse IgG (Sigma, USA) was used as a secondary antibody at a 1:50 dilution, and slides were viewed at a magnification of ×40 on a fluorescence microscope (Leica, Germany) [47].

The positive cell clones were subcloned three times by limiting dilution method. Selected clones were cultured in the peritoneal cavities of pristane (Sigma, USA)-primed BALB/c mice to obtain ascites.

The mAb titer was determined by indirect ELISA as described above and Ig subtypes of them were determined using the Mouse MonoAb-ID Kit (HRP) (Invitrogen, Carlsbad, CA, USA) according to the manufacturer's instructions. This test identified IgG1, IgG2a, IgG2b, IgG3, IgA and IgM subtype classes, while κ and λ light chains were determined using monospecific rabbit polyclonal antibodies (Pabs).

### Determination of epitopes by phage-displayed random peptide library

The Ph.D.-12™ Phage Display Peptide Library Kit was purchased from New England BioLabs Inc.. The dodecapeptide library consists of 2.7 × 10^9 ^electroporated sequences (1.5 × 10^13 ^pfu ml^-1^). All mAbs were purified from ascites of mice inoculated with the hybridoma cells secreting antibody by affinity chromatography using rProtein G (Sigma, USA) according to the manufacturer's instructions, and the concentration of purified antibody was determined by the Bradford Protein Assay Kit (http://www.beyotime.com/CompatibilityChartForBradfordKit.Pdf).

Three successive rounds of biopanning were carried out according to the manufacturer's instruction manual. Briefly, one well of a 96-well microtiter plate was coated with 15 μg of purified mAb in coating buffer (0.1 M NaHCO_3_, pH 8.6), followed by blocking with blocking buffer (0.1 M NaHCO_3_, pH 8.6 and 5 mg ml^-1 ^BSA) for 2 h at 4°C. About 1.5 × 10^11 ^pfu (4 × 10^10 ^phages, 10 μl of the original library) were added to the well and incubated for 1 h at room temperature by gentle shaking. Unbound phages were removed by successive washings with TBS buffer (50 mM Tris-HCl, pH 7.5; 150 mM NaCl) containing gradually increased concentrations (0.1%, 0.3%, and 0.5%) of Tween-20, and bound phages were eluted with elution buffer (0.2 M Glycine-HCl, pH 2.2) containing 1 mg ml^-1 ^BSA. The eluted phages were amplified in early-log *E. coli *ER2738 strain cells. After three rounds of biopanning, ten individual phage clones were selected and assayed for target binding by sandwich ELISA as described by the manufacturer's instructions. Briefly, 96-well microtiter plates were coated overnight with 2 μg of mAb or irrelevant control mAb (anti-porcine IFN-γ mAb, Sigma, USA). After 2 h of blocking with blocking buffer at 4°C, phage clones were added to the wells (2 × 10^11 ^pfu in 100 μl per well) and incubated with agitation for 2 h at room temperature. Bound phages were subjected to reaction with HRP-conjugated anti-M13 antibody (Pharmacia, USA) for 2 h at room temperature, followed by color development with substrate solution containing *o*-phenylenediamine (OPD).

The DNA inserts displayed by ELISA-positive phage clones were sequenced with the 96 gIII sequencing primer: 5'-TGAGCGGATAACAATTTCAC-3' as described by the manufacturer's instructions (New England BioLabs Inc.).

### Identification of the displayed epitopes by WB using expressed polypeptides

A series of complementary oligonucleotides (Table 1) encoding for wild-type and truncated versions of the motifs LTATTEK and VVDGPETKEC were synthesized, annealed and cloned into the *EcoR *I/*Sal *I sites of the prokaryotic expression vector pMAL™-C2x (New England Biolabs, Inc., USA), resulting in a group of recombinant plasmids. The *E. coli *TB1 cells with the recombinant plasmids were induced by IPTG up to 0.5 mM to produce recombinant MBP-fusion polypeptides, then identified the serial of polypeptides expression by WB using anti-MBP-tag mAb (New England Biolabs, Inc., USA). WB was performed as described above.

**Table 1:** Oligonucleotide primers used to assemble short DNA fragments coding for wild-type and truncated epitope sequences

Designations of primers	Sequences of primers	Sequences of coded peptides (designations)
Cp-1-F	5'-AATTCctcaccgccaccacggaaaaa*TAA*G-3'	LTATTEK (Cp-1)
Cp-1-R	5'-TCGACTTAtttttccgtggtggcggtgagG-3'	
Cp-2-F	5'-AATTCaccgccaccacggaaaaa*TAA*G-3'	TATTEK (Cp-2)
Cp-2-R	5'-TCGACTTAtttttccgtggtggcggtG-3'	
Cp-3-F	5'-AATTCctcaccgccaccacggaa*TAA*G-3'	LTATTE (Cp-3)
Cp-3-R	5'-TCGACTTAttccgtggtggcggtgagG-3'	
Cp-4-F	5'-AATTCgccaccacggaaaaa*TAA*G-3'	ATTEK (Cp-4)
Cp-4-R	5'-TCGACTTAtttttccgtggtggcG-3'	
Cp-5-F	5'-AATTCctcaccgccaccacg*TAA*G-3'	LTATT (Cp-5)
Cp-5-R	5'-TCGACTTAcgtggtggcggtgagG-3'	
Dp-1-F	5'-AATTCgtggttgatggtccggagaccaaggaatgt*TAA*G-3'	VVDGPETKEC (Dp-1)
Dp-1-R	5'-TCGACTTAacattccttggtctccggaccatcaaccacG-3'	
Dp-2-F	5'-AATTCgttgatggtccggagaccaaggaatgt*TAA*G-3'	VDGPETKEC (Dp-2)
Dp-2-R	5'-TCGACTTAacattccttggtctccggaccatcaacG-3'	
Dp-3-F	5'-AATTCgtggttgatggtccggagaccaaggaa*TAA*G-3'	VVDGPETKE (Dp-3)
Dp-3-R	5'-TCGACTTAttccttggtctccggaccatcaaccacG-3'	
Dp-4-F	5'-AATTCgatggtccggagaccaaggaatgt*TAA*G-3'	DGPETKEC (Dp-4)
Dp-4-R	5'-TCGACTTAacattccttggtctccggaccatcG-3'	
Dp-5-F	5'-AATTCgtggttgatggtccggagaccaag*TAA*G-3'	VVDGPETK (Dp-5)
Dp-5-R	5'-TCGACTTActtggtctccggaccatcaaccacG-3'	
Dp-6-F	5'-AATTCggtccggagaccaaggaatgt*TAA*G-3'	GPETKEC (Dp-6)
Dp-6-R	5'-TCGACTTAacattccttggtctccggaccG-3'	
Dp-7-F	5'-AATTCgtggttgatggtccggagacc*TAA*G-3'	VVDGPET (Dp-7)
Dp-7-R	5'-TCGACTTAggtctccggaccatcaaccacG-3'	

*Notes*: Nucleotides introduced to form the overhanging ends of *EcoR *I and *Sal *I, and the TAA stop codon are shown in italic letters.

### Detection of the reactivity of the epitopes with WNV/JEV-positive equine serum

To verify whether the epitopes could be detected by WNV/JEV-positive serum, the polypeptides MBP-Cp-2 (MBP fusion containing peptide of Cp-2) and MBP-Dp-1 (MBP fusion containing peptide of Dp-1) were subjected to reaction with WNV/JEV-positive equine serum by WB. WB was performed as described above, but the primary antibody was WNV/JEV-positive equine serum and HRP-conjugated rabbit anti-equine secondary antibodies (LICOR Biosciences) were used [47]. The same test was also performed using WNV-negative equine serum.

### Homology and cross-reactivity analysis

To investigate the homology of the two epitopes to equivalent sequences of flaviviruses, alignments of sequences from homologous regions of 18 WNV NS1 (including 3 Kunjin virus strains) were completed by using the DNASTAR Lasergene program (Windows version; DNASTAR Inc., Madison, WI). Alignment analysis was also performed between the identified epitopes and other associated flavivirus strains, including the members of JEV serocomplex, and another three antigenically related flaviviruses, DENV (type 1-4), YFV and TBEV, the factors of isolation time and geographical location of all strains were considered.

## Competing interests

The authors declare that they have no competing interests.

## Authors' contributions

DLW designed the experiment. ECS and JNM carried out most of the experiments and ECS wrote the manuscript. RAL supplied the equine serum against WNV. YHY supplied the results of IFA. ZGB supplied the WNV C gene. TY, JZ, HWG, YLQ, LFW and NHL participated part of experiments. DLW and LFW revised the manuscript. All authors read and approved the final manuscript.
